# Natural personal care products—analysis of ingredient lists and legal situation

**DOI:** 10.1186/s12302-016-0076-7

**Published:** 2016-03-24

**Authors:** Ursula Klaschka

**Affiliations:** University of Applied Sciences Ulm, Prittwitzstr. 10, 89075 Ulm, Germany

**Keywords:** Classification and labeling, Cosmetics, Hazardous substances, Natural substances, Personal care products

## Abstract

**Background:**

Many natural substances are classified as dangerous substances according to the European regulation on classification and labelling. Are they used in natural personal care products today? One hundred ingredient lists were analyzed to find this out.

**Results:**

All products with natural substances contained dangerous natural substances or they contained natural substances, for which the information about their classification as dangerous substances is not available. 54 natural substances quoted in the ingredient lists were found to be classified, with 37 substances being classified due to hazardous effects for skin and eyes. However, the most frequently used natural substances are not classified as dangerous. Natural substances are multi-constituent compounds, leading to two main problems in personal care products: the potential interactions of a multitude of substances and the fact that dangerous constituents are not disclosed in the ingredient lists. For example, the fragrance allergens citral, farnesol, limonene, and linalool are frequent components of the natural substances employed. In addition, 82 products listed allergenic fragrance ingredients as single substances in their ingredient lists. Recommendations for sensitive skin in a product’s name do not imply that the ‘26 fragrance allergens’ are omitted. Furthermore, 80 products listed ‘parfum’/‘aroma’, and 50 products listed ethanol.

**Conclusions:**

The data show that the loopholes for natural substances and for personal care products in the present European chemical legislation (e.g. the exception for classification and labelling of cosmetic products and the exception for information transfer in the supply chain) are not in line with an adequate consumer and environmental protection.

## Background

Many consumers consider natural products as an alternative with a benefit for their healthy lifestyle and grant them a bonus as ‘greener’ or ‘safer’ compared to conventional products. There is also a new trend that consumers tend to avoid certain compounds in products [1], e.g. parabens, endocrine disruptors such as phthalates (http://www.bund.net/toxfox), or fragrances [2, 3]. There are products on the market that tout with labels that they are free of these ingredients and in addition free of preservatives, PEG emulsifiers, paraffin, mineral oil, silicon, or micro plastic material. Consumers might assume that these personal care products contain less chemicals of concern, although they usually do not know the properties and concentrations of the ingredients. Many consumers are not aware that the allergenic potential of the fragrance ingredients of these products might not be smaller. These market trends reflect the concern of consumers about dangerous ingredients in personal care products and the difficulty for them to make the right choice [4]. Consumers might be misled about the product safety from the attractive containers, appealing messages promised by the cosmetic industry and the lack of warning symbols [5–7]. These messages tend to make consumers trust the products and make them less aware that they might contain dangerous substances. There are no warning symbols on personal care products, because they are exempt from the obligation to classify and label products that contain hazardous substances above the thresholds for classification [8]. If, however, PCPs were classified and labelled in accordance with the criteria of the European regulation on classification, labelling and packaging of substances and mixtures (CLP-regulation [8]), many of them would require hazard pictograms on the label [9]. Consumers can use other information sources, such as the ingredient lists, which have to be printed on the containers of personal care products according to the European cosmetics regulation [10]. Most natural substances can be recognized in these lists by the binomial Latin names of the organism of origin, e.g. *Rosa damascena* extract is an extract of the Damask rose. The Latin names do not need to be complemented by common names. Names like *Rosa* (rose), *Lavandula* (lavender) or *Rosmarinus* (rosemary) are similar to the common names and easily understood, whereas *Urtica urens* (stinging nettle), *Prunus armeniaca* (apricot) or *Simmondsia chinensis* (jojoba) are probably not familiar to most of the consumers, just like the names of most chemically synthesized ingredients. Some authors consider the use of natural substances as ingredients in personal care products since antiquity as unspoken indication for their safety [11]. However, the traditional use of chemicals does not guarantee that they are safe. Some examples of toxic substances, which were used in the past or have been used for a long time, are lead salts, antimony and copper (as ingredients in decorative cosmetics in Ancient Egypt) or henna (as skin and hair dye). Many natural substances that are employed in personal care products are classified as hazardous substances according to the CLP-regulation [8]. A recent study showed that out of 1358 natural substances in the ‘International Nomenclature of Cosmetic Ingredients’ (INCI list or Common Ingredients Glossary [12]) 369 natural substances are classified as hazardous substances [13]. 257 substances are classified due to their negative effects on human health (226 due to their effects on skin and eyes), and 182 substances are classified due to their negative effects on the aquatic environment. 53 natural substances in the INCI list are classified as carcinogens, mutagens and substances toxic to reproduction. The point of departure of the present study was to find out whether these substances were really used in so-called natural personal care products in today’s retail market in Europe.

## Results and discussion

### Big variety of natural substances used in natural cosmetic products

#### Most natural substances come from food and medicinal plants

The ingredient lists of a random sample of 100 natural personal care products by ten manufacturers comprised 231 different natural substances. These natural substances derived from plants, with the exception of cera alba (beeswax), mel (honey), mel extract, royal jelly extract, Xanthan gum (polysaccharide secreted by the bacterium *Xanthomonas campestris*) and shellac (resin secreted by the female lac bug *Kerria lacca*). Many of the natural substances listed originated from food plants (Table 1a), some of which are flavoring plants, such as lemongrass, rosemary, vanilla, or ginger. Other natural substances derive from parts of food plants that are not edible, such as *Avena sativa* straw extract, *Juglans regia* shell powder, *Prunus armeniaca* kernel oil, *Prunus domestica* seed oil, *Ribes nigrum* leaf extract, *Rubus idaeus* seed oil, *Vitis vinifera* leaf or seed extract. Other natural substances derive from plants known for their pharmaceutical uses (Table 1b).

**Table 1:** Examples of natural substances declared on the ingredient lists of a random sample of 100 natural personal care products, which were derived from plants used as food (a) and as pharmaceutical plants (b)

a) *Arachis hypogaea* (peanut), *Avena sativa* (oat), *Camellia sinensis* (tea), *Brassica napus* (rape), *Carica papaya* (Papaya), *Chondrus crispus* (Irish moss), *Citrus aurantium amara* (bitter orange), *Citrus aurantium dulcis* (orange), *Citrus aurantifolia* (key lime), *Citrus limon* (lemon), *Citrus medica limonum* (citron), *Cocos nucifera* (coconut), *Coffea arabica* (coffee plant), *Cymbopogon citratus* (lemongrass), *Daucus carota* (carrot), *Euterpe oleracea* (Açaí palm), *Foeniculum vulgare* (fennel), *Glycine soja* (soy plant), *Glycyrrhiza glabra* (licorice), *Helianthus annuus* (sunflower), *Juglans regia* (walnut), *Macadamia ternifolia* (macadamia), *Mangifera indica* (mango), *Manihot utilissima* (cassava), *Mentha piperita* (peppermint), *Olea europaea* (olive), *Oryza sativa* (rize), *Panax ginseng* (ginseng), *Panicum miliaceum* (common millet), *Passiflora edulis* (passion fruit), *Paullinia cupana* (Guarana) *Persea gratissima* (avocado), *Prunus amygdalus dulcis* (almond), *Prunus domestica* (plum), *Prunus persica* (peach), *Punica granatum* (pomegranate), *Pyrus cydonia* (quince), *Ribes nigrum* (currant), *Rosmarinus officinalis* (rosemary), *Rubus idaeus* (raspberry), *Saccharum officinarum* (sugar cane), *Sesamum indicum* (sesame), *Theobroma cacao* (cocoa), *Manihot esculenta* (tapioca), *Triticum vulgare* (common wheat), *Vaccinium macrocarpon* (cranberry), *Vaccinium myrtillus* (blueberry), *Vanilla planifolia* (vanilla), *Vitis vinifera* (grape vine), *Zea mays* (maize), *Zingiber officinale* (ginger)
b) *Arnica montana* (arnica), *Calendula officinalis* (calendula), *Chamomilla recutita*, (matricaria, chamomile), *Chondrus crispus* (Irish moss), *Eucalyptus globulus* (bue gum), *Glycyrrhiza glabra* (licorice), *Hypericum perforatum* (St John’s wort), *Melissa officinalis (*lemon balm), *Mentha piperita* (peppermint), *Ricinus communis* (castor oil), *Salvia officinalis* (common sage)

#### Many names of natural substances on the ingredient lists do not appear in the official list for cosmetic ingredients

The data search was impeded, because only 77 names of natural substances were the correct mandatory names of the INCI list, and 154 names did not show up in the INCI list (Table 2), although several companies refer to the INCI/CTFA (list by the Personal Care Products Council) in the heading of the ingredient lists. However, we could derive the identity of 71 substances with the non-INCI name by the description in the INCI list about the origin of the respective natural substance. For example, the INCI name is ‘*Sesamum indicum* oil’, and the further explanation in the INCI list says ‘*Sesamum indicum* oil is the oil obtained from the seed of sesam, *Sesamum indicum*, Pedaliaceae…’. If a company writes, ‘*Sesamum indicum* (sesame) seed oil’ in the ingredient list, it is clear that this means the same natural substance as the correct INCI name ‘*Sesamum indicum* oil’. In such cases, the names used by the companies give more information about the natural substance (common name of the plant and part of the plant used) compared to the INCI name. This wording is in line with the legal requirements in the USA and also acceptable in the EU. However, no INCI name could be derived for 83 natural substances listed in the ingredient lists (examples in the footnote in Table 2). Most substances that are not in the INCI list were quoted only in one or two of the 100 ingredient lists, exceptions were: *Magnolia officinalis* bark extract (on eight products by two companies), *Rosa damascena* flower water (on seven products by four companies)*, Punica granatum* seed oil (on five products by five companies), *Sambucus nigra* root extract (on four products by one company). These substances were not analyzed further here, as no distinct identity of the substance with CAS or EC number and its classification could be attributed. Problems with the inconsistency of the names of natural substances had been described in a previous study [13]. Furthermore, taxonomic plant names did not correspond to the INCI names, when old taxonomic names were employed, e.g. *Triticum vulgare* is the old name in the INCI list, *Triticum aestivum* is the new taxonomic name. This was already noted in the cosmetic ingredient review (CIR) on plant-derived fatty acid oils [11]. Furthermore, there were several typing errors in the ingredient lists: e.g. ‘*Cupressus semipervirens’* instead of ‘*Cupressus sempervirens*’, or ‘*Ruscus aculateus’* instead ‘*Ruscus aculeatus’,* ‘*Lavendula augustifolia* flower extract’ instead of ‘*Lavandula angustiflia* flower extract’.

**Table 2:** The ingredient lists of 100 natural personal care products contained 231 different natural substances

Number of natural substances	Correct INCI names	Incorrect INCI names		Examples^b^
231	77	154		
		INCI names derived 71	No INCI names derived 83^a^	
Classified due to health and environmental hazards	14	24	Number unkown	*Chamomilla recutita* extract *(12)*(H304 H315 H317 H412), *Citrus aurantium dulcis oil (5)* (H226 H304 H315 H317 H400 H410), *Citrus medica limonum* oil *(2)(*H226 H304 H315 H317 H400 H410), *Daucus carota* juice *(1)* (H226 H304 H317 H319 H411), *Lavandula angustifolia* oil *(5)* (H304 H315 H317 H412), *Melissa officinalis* oil *(1)* (H315 H317 H318 H412), *Pelargonium graveolens* oil *(1)* (H304 H315 H317 H318 H412), *Ribes nigrum* extract *(1)* (H304 H317 H411), *Rosa damascena* extract *(3)* (H226 H315 H317 H318 H341 H351 H412), *Rosmarinus officinalis* extract *(5)*(H226 H304 H317 H373 H411), *Salvia officinalis* extract *(5)* (H226 H304 H315 H317 H373 H400 H410), *Triticum vulgare* germ extract *(1)* (H317), *Urtica urens* extract *(1)* (H360)
Classified due to physical hazards only	4	12	Number unkown	*Camelia sinensis* extract *(10)* (H225), *Coffea arabica* extract *(1)* (H226), *Ginkgo biloba* extract *(1)* (H226), *Panax ginseng* extract *(2)* (H226), *Paullinia cupana* extract *(2)* (H226), *Prunus armeniaca* kernel oil *(6)* (H226), *Sambucus nigra* extract *(1)* (H225), *Tilia cordata* extract *(1)* (H226)
Not classified	31	24	Number unkown	*Aloe barbadensis* extract *(10)*, *Butyrospermum parkii* (Shea) butter *(29)*, *Calendula officinalis* extract *(7)*, *Cocos nucifera* oil *(5)*, *Helianthus annuus* seed oil *(33)*, *Hypericum perforatum* extract *(1)*, *Olea europaea* oil *(21)*, *Prunus amygdalus* *dulcis* extract *(3)*, *Quercus robur* extract *(1)*, *Ricinus communis* oil *(3)*, *Sesamum indicum* oil *(12)*, *Simmondsia chinensis* oil *(35)*, Xanthan gum *(50)*
Not in the C & L inventory	28	11	Number unkown	*Aloe barbadensis* gel *(17) Calendula officinalis* oil *(2)*, *Euphrasia officinalis* extract *(1)*, *Hippophae rhamnoides* oil *(7), Oenothera biennis* oil *(6), Prunus amygdalus dulcis* oil *(23)*, *Rosa canina* fruit oil *(3)*, *Salvia officinalis* oil *(3)*, *Triticum vulgare* germ oil *(4)*, *Vitis vinifera* seed oil *(4)*

Examples are given with the H-phrases (^b^explanations in the footnote) as taken from the C & L inventory. The numbers in italics and brackets indicate the number of products where the respective natural substance was named on the ingredient lists

^a^names that are not in the INCI list, e.g. *Aesculus hippocastanum* seed extract *(1)*, *Brassica oleracera italica* seed oil *(1)*, *Euphorbia cerifera* (candelilla) wax *(1)*, *Euterpe oleracea* fruit oil *(5)*, *Fusanus spicatus* wood oil *(1)*, *Hamamelis virginiana* (witch hazel) leaf water *(5)*, *Magnolia officinalis* bark extract *(8), Mesembryanthemum crystallinum* extract *(1)*, *Prunus spinosa* flower extract *(3)*, *Punica granatum* seed oil (5), *Ribes nigrum* (currant) leaf extract *(2), Rosa damascena* flower oil *(4)*, *Rosa damascena* flower water *(7)*

^b^Explanations of H-phrases: H225 Highly flammable liquid and vapor, H226 Flammable liquid and vapor, H304 May be fatal if swallowed and enters airways, H314 Causes severe skin burns and eye damage (skin corrosive), H315 Causes skin irritation, H317 May cause an allergic skin reaction, H318 Causes serious eye damage, H319 Causes serious eye irritation, H341 Suspected of causing genetic defects, H351 Suspected of causing cancer, H360 May damage fertility or the unborn child, H373 May cause damage to organs through prolonged or repeated exposure, H400 Very toxic to aquatic life, H410 Very toxic to aquatic life with long lasting effects, H411 Toxic to aquatic life with long lasting effects, H412 Harmful to aquatic life with long lasting effects

#### Dangerous natural substances are listed in most ingredient lists

A high number of natural compounds is bioactive [14–18], and it was shown previously that many natural substances in the INCI list are classified as dangerous substances [13]. The issue in the present study was to find out whether some of these substances are also used in today’s natural cosmetic products. This was clearly the case, as all ten companies analyzed here listed natural substances classified as dangerous in the ingredient lists of their products (Table 2). 148 natural substances had a clear substance identity with CAS or EC number. Out of these 54 are classified as dangerous substances according to the CLP-regulation, with 37 as classified due to negative effects on skin and eyes (e.g. H314, H315, H317, H318, H319). Some are also carcinogens, mutagens and substances toxic to reproduction (CMR substances), classified with H341, H351 or H360. Glycidol and glycidol fatty acid esters are impurities that can occur in refined vegetable oils and may be carcinogenic, but the CIR assumes that absorption through the skin would be very low and hence ‘does not pose a significant hazard’ [11]. Some oils, which are used in aerosolized products, can be inhaled and exert effects on the lungs [11]. *Lavandula angustifolia* oil was described to have estrogenic effects (prepubertal gynecomastia in boys), and anti-androgenic effects [19]. There were 65 products with at least one classified natural substance on the ingredient list, and there were 34 products, that contained only natural substances with no publically available information about their classifications. One product did not contain any natural substances. This means that there is no product at all among the products containing natural substances, which for sure does not contain a dangerous natural substance. Among the names, which were not correct INCI names, there were 24 natural substances that are classified as toxic for the human health. And here were 39 natural substances with identified INCI names, which did not appear in the C & L inventory and for which no information about the classification and labelling was publicly available.

#### Few natural substances are registered according to REACH

According to the European regulation on registration, evaluation, authorization and restriction of chemicals [REACH regulation [20], Art. 2(7b) and Annex V (8)] natural substances, which are classified and labelled as dangerous substances need to be registered. However, only the following natural substances, which appeared in the present study, were registered according to REACH: *Mentha piperita* water and various preparations of *Citrus aurantium dulcis, Citrus medica limonum* and *Citrus aurantium amara*. Annex IV of the REACH regulation is the list of substances, which do not need to be registered. This is relevant for three oils in the 100 natural personal care products analyzed here: sunflower oil (*Helianthus annuus*), soybean oil (*Glycine max*), and castor oil (*Ricinus communis*). These oils are not classified in the C & L inventory.

### The frequencies and the amounts used in the products vary widely depending on the product type and the brand

The ten companies reported natural substances in very diverging frequencies (Table 3). The company with the highest frequency listed 99 times natural substances in their ten products selected, compared to 43 times in the company with the lowest frequency. Manufactures of expensive products tend to specify more natural substances on their ingredient lists than their competitors selling products at low prices. Xanthan gum was the natural substance most frequently listed (50 times) followed by *Simmondsia chinensis* (Jojoba) oil (35), *Helianthus annuus* seed (sun flower) oil (33), *Butyrospermum parkii* (Shea) butter (29), Cera alba (bees wax) (23), *Prunus amygdalus dulcis* (almond) oil (23), *Olea europaea* (olive) oil (21), *Aloe barbadensis* gel (17), *Glycine soja* (soybean) oil (17), *Chamomilla recutita* extract (12), *Sesamum indicum* oil (12). Among these predominately-used natural substances only *Chamomilla recutita* extract is classified as dangerous for human health and the environment. *Prunus amygdalus dulcis* oil and *Aloe barbadensis* gel were not yet in the C & L inventory. Interestingly, 131 out of the 231 natural substances declared on the 100 personal care products in this study appeared only once by one company. *Triticum aestivum* germ oil was used by two companies in four products, whereas it was thought previously to have no reported uses [11]. Xanthan gum, the most frequently named natural substance, is used in most products only in minor amounts for emulsion stabilizing and viscosity controlling; jojoba oil is used in most products in considerable amounts as emollient. In a comprehensive study on the use of oils in cosmetic products, CIR had reported that *Butyrospermum parkii* (shea) butter was the most frequently used oil, followed by *Helianthus annuus* (sunflower) seed oil. *Prunus amygdalus dulcis* (sweet almond) oil, *Olea europaea* (olive) fruit oil, and *Glycine soja* (soybean) oil are also among the most frequently applied oils in personal care products according to the study by CIR. These oils have use concentrations in the products of up to 100 % [11]. The pattern of oils used in the 100 products analyzed here corresponds rather well with the data by CIR. This shows that the present small data collection can give a good indication about the general usage of natural substances.

**Table 3:** The ten companies of the present study and their brand specific selection of natural substances

All natural substances that were indicated at least four times in the 100 ingredient lists were ordered according to their frequencies in all ingredient lists. The color shades visualize the number of listings in the ingredient lists

#### The INCI list suggests various functions in cosmetic products for each substance

The main functions of the natural substances reported here as recommended in the INCI list are skin conditioning (55 substances), emollient (51 substances) and tonic (48 substances). Functions like astringent (28 substances), soothing (26 substances) and masking (20 substances) are also relatively frequent properties of the natural substances employed in these products. Functions that imply physiological activity are not very frequent, e.g. antidandruff (4) or antimicrobial (8).

#### Natural substances are multi-constituent substances of variable composition

Natural substances belong to the group of so called ‘UVCB substances’, which are chemicals with ‘unknown or variable composition, complex reaction products or biological materials’ [20]. Growth conditions of the organism, preparation methods, storage conditions and other parameters affect the composition of a natural substance. These are reasons, why proportions of constituents can vary between producers, leading to different information in their safety data sheets. The toxicity of natural substances does not only depend on the plant of origin and the preparation method, but also on the quality of the products and the properties of contaminations. Potential interactions of ingredients—in the products themselves as well as during application—should therefore be a big issue for products containing natural substances, especially of products applied to the skin where additional reactions can be triggered by UV radiation. The multiple components of natural substances can also interact with the synthetic ingredients. All 100 products had on average 20.6 ingredients (natural and other) listed (minimum 1 and maximum 38). 26 of those products had more natural substances (according to the definition used here) than other ingredients declared on the label. The products listed on average 7.3 natural substances (minimum zero, maximum 20). These numbers would increase considerably if also chemically modified natural substances were counted. It is not possible to estimate how many different chemical constituents might be present in such cosmetic products.

#### Natural substances can exert different effects when applied to the skin or when ingested via food

Food application results in much larger systemic doses than application as personal care products for most natural ingredients considered here. Uptake via food will encounter metabolic pathways developed over the course of evolution and affect mainly internal organs [21]; whereas the prevalent potential toxic effects of external application to the skin via cosmetic products are effects like contact allergy, skin irritation or phototoxicity. These effects should not be underestimated. For example, the importance of skin exposure as a route for induction of sensitization to peanut is still under debate. It was a pragmatic decision that peanut oil below a protein level of 0.5 ppm is considered safe for cosmetic use, as there are insufficient data to define a safe level of skin exposure in the non-sensitized population [22]. Four creams in this study contained peanut oil (*Arachis hypogaea* oil) without special information about peanut allergy. Ruden and Hansson [23] state that data needed for hazard assessment and classification for skin and eye irritation are fundamental for safe handling at the workplace. I support this postulation and go even further: I plead that data needed for hazard assessment and classification for hazardous skin and eye effects should be made transparent for the consumer of products that are used every day like personal care products. In addition, substances applied on the body surface can also exert systemic actions, as substances can be taken up readily [24]. For example, the maximum daily exposure of humans by coumarin, which was specified on 18 products in the present study, has been estimated to be in the same order of magnitude from the application of personal care products compared to the uptake of food [25]. In addition to food and personal care products, also pharmaceuticals, air fresheners, washing and cleaning products may contain natural substances. It is important to consider the sum of all exposure routes and all possible sources for a realistic assessment of dangerous substances. The exposure via personal care products is not negligible. More than three billon personal care products are sold per year in Germany [26]. This number shows the wide distribution of these products and their relevance for consumers and the environment.

### Only partly ‘free of … ‘

Table 4a shows the substances that are dangerous substances or discussed as substances of concern [1, 27–29] that were not found in any of the 100 ingredient lists. This shows that companies producing natural cosmetics seem to take over responsibility and renounce these hazardous substances in comparison with conventional products [30]. However, it cannot be excluded that the products contain such substances without listing them on the labels: In 2000, Rastogi found that the declarations of preservatives in the ingredient lists in 45 % of the analyzed skin creams were wrong [31]. Dodson and his group had analytically detected fragrance compounds, diethanolamine, methylparaben and various phthalates in ‘alternative’ cosmetic products that were not listed on the labels [27]. These substances can find their way into the products as proper ingredients, but also as solvents, contaminants, additives or preservatives of the raw material.

**Table 4:** All cosmetic products analyzed did not catalogue substances in (a), whereas substances in (b) were listed in the ingredient lists of several products (number of products given in brackets)

(a) Benzalkonium chloride, benzophenone-3 (BP-3), biguanides, bisphenol A, 2-butoxyethanol, butylphenyl methylpropional, carbomer, chloroacetamide, cocamidopropyl betaine, cyclomethicone, cyclosiloxanes, dieethanolamine, formaldehyde, formic acid, glutardialdehyde, glycolester, hydrochloric acid, hydrogenperoxide, isothiazolones, lauryl alcohol, 2,2-methoxyethoxyethanol, monoethanolamine, musk fragrances, nanoparticles, octinoxate (octyl methoxycinnamate, phenylenediamine, octyl dimethyl PABA (*p*-aminobenzoic acid), parabens, PHMB (Polyhexamethylenbicyanoguanide), phenylenediamine, phthalates, propanol, propylene glycol, quarternary ammonium compounds, sodium hypochlorit, triclosan
(b) Alcohol (ethanol) (50), aluminium salts (3), benzyl alcohol (14), benzyl benzoate (14), glycerol (70), gold (3), lactic acid (13), silver (3), sodium benzoate (11), zinc compounds (12)

Table 4b shows the substances that were listed in the ingredient lists. Every second product contained alcohol (ethanol), mostly among the first five substances in the ingredient lists, indicating a major percentage in the products. Glycerol appeared on 70 natural products, also mostly as one of the first substances listed. Interestingly, some companies do not employ alcohol or glycerol in any products, whereas other companies employ ethanol or glycerol in almost every product. Three products contain gold and silver. Both metals are not indicated as nano ingredients. Two products contain titanium dioxide and iron oxides. Three products contain aluminum salts. There are 12 products that contain zinc compounds: ten are products for daily use (deodorants, mouthwash, lip balm, day cream), and two indicate a therapeutic benefit in their names (‘anti pimples’, ‘vital serum’). Benzyl alcohol, benzyl benzoate and sodium benzoate are preservatives with sensitizing potential [32]. Benzyl alcohol is at the same time a fragrance (see Table 5). There is no doubt that preservatives might be needed even more in personal care products that contain natural substances than in conventional products to reduce growth of microorganism, however the consumer might not expect preservatives with sensitizing.

The ingredient lists of rinse-off products (shampoo, soap, shower gel) and leave-on products (creams and deodorants) were analyzed separately and led to the following results: Rinse-off products contained on average 6.2 natural substances (on average 1.8 were not the correct INCI names), whereas leave-on products contained on average eight natural substances per product, (four of which were not the correct INCI names). There is no relevant difference between the number of alcohol containing rinse-off products and leave-on products. The differences of the compositions between all rinse-off and all leave-on products are smaller than the differences between companies. The compositions of products within each product type showed a substantial variability, concerning the presence of ethanol, glycerol and ‘parfum’.

### Parfum and fragrances as frequent ingredients

#### The vast majority of the products contain ‘parfum’

‘Parfum’ in the ingredient list stands for up to several hundred fragrance ingredients which are not disclosed to the consumer and often not even to the down-stream users in the supply chain. Especially some of the chemically synthetized pure constituents proved to be of toxicological and ecotoxicological relevance. Some are known sensitizers for allergic contact dermatitis [33–35]. Nitro- and polycyclic musk compounds have been studied with great attention since they were detected in considerable concentrations in the environment [24, 36, 37]. Musk xylene, known for its estrogenic potential, is now a substance of very high concern (SVHC) according to REACH [20] and included in the authorization list. In 2015, also the fragrance Karanal (5-sec-butyl-2-(2,4-dimethylcyclohex-3-en-1-yl)-5-methyl-1,3-dioxane, EC 413-720-9) has been suggested for the candidate list of SVHCs (http://echa.europa.eu/web/guest/candidate-list-table). Karanal is used in perfumes, is very persistent and has a high potential for bioaccumulation. SVHCs must be authorized before they are placed on the market or used, unless they are used in cosmetic products ([20] Art. 56 (5)). With this exception for cosmetic products, it must be assumed, that the authorization will only affect other uses, but not perfume.

71 natural cosmetic products contained ‘parfum’. (15 indicated that the ‘parfum’ consisted of essential oils.) ‘Aroma’ was listed on nine products that did not contain ‘parfum’, this means that in total 80 products declared either ‘parfum’ or ‘aroma’.

**Table 5:** The ‘26 fragrance allergens’ in the ingredient lists in the order of frequency of appearance

INCI-Name CAS no	Allergic potential	Classification and	Labelling	Number of products with this fragrance in the ingredient list
D-Limonene 5989-27-5	III	H226, H315, H317, H400, H410		71
Linalool 78-70-6	III	H315, H319		70
Geraniol 106-24-1	II	H315, H317, H318		54
Citronellol 106-22-9	II	H315, H317, H319		45
Citral 5392-40-5	II	H315, H317		44
Farnesol 602-84-0	I	H315, H317, H319		19
Coumarin 91-64-5	II	H302, H317		18
Eugenol 97-53-0	II	H317, H319		16
Benzyl alcohol 100-51-6	III	H302, H322		14
Benzyl benzoate 120-51-4	III	H302, H411		13
Benzyl salicylate 118-58-1	III	H317, H411		7
Cinnamyl alcohol 104-54-1	II	H317		2
Isoeugenol 97-54-1	I	H302, H312, H315, H317, H319		2
Cinnamal 104-55-2	I	H312, H315, H317, H319		2

The fragrance allergens that were not mentioned on any product are not listed here. Column 2 shows the allergenic potential according to [39] [Group I: important allergens, Group II: clearly allergenic, but less important in terms of sensitization frequency, group III: (extremely) rare sensitizers or even non-sensitizers]. Column 3 and 4 show the classification and labelling by the majority of notifiers or the harmonized classification if available in the C & L inventory

#### The vast majority of the products contain allergenic fragrance ingredients

According to the cosmetics regulation, 26 potential allergenic fragrance substances have to be indicated on the product containers if they are present above 0.001 percent in ‘leave-on’ products or above 0.01 percent in ‘rinse-off’ products [10, 38]. These 26 potential allergenic fragrance ingredients are chemically defined constituents. All ten manufacturers used several of them. 82 natural personal care products reported at least one of the ‘26 fragrance allergens’. On average, the natural products analyzed declared 3.8 of the ‘26 fragrance allergens’, with a maximum of 11 per product. Leave-on products listed on average even more of the ‘26 fragrance allergens’ per product (3.4 on average) compared to rinse-off products (2.6 on average). On 58 products, it said that these fragrances originated from natural sources. It must be emphasized here, that the allergenic potential of a chemical is independent of whether a substance is of natural origin or synthetic. It depends on chemical structure, concentration, purity and interactions with other compounds.

Table 5 shows which of the potential allergens were used preferentially in the 100 natural cosmetic products. The most frequently indicated fragrance allergens are limonene and linalool, two allergens, which are rare sensitizers themselves, but their oxidation products are potent sensitizers [40–42]. The combined exposure to a variety of allergenic fragrances can result in a considerable risk, even if the single fragrances have only a low allergenic potential [43].

#### Allergenic fragrance substances are natural constituents of many natural substances employed, leading to an aggregate exposure, but they are not disclosed by name in the ingredient lists

It is important to note that natural substances are multi-constituent substances, which may contain some allergenic fragrances as natural constituents (Table 6; [44]). A consumer who wants, for example, to avoid exposure to limonene should not use products that contain *Citrus medica limonum* oil, *Cupressus sempervirens* oil, *Cymbopogon martini* oil, *Eucalyptus globulus* oil, *Rosmarinus officinalis* leaf oil or other natural substances. This means that these consumers have a difficult task, because they should know the relevant constituents of natural substances. The fragrance constituents of natural ingredients do not appear on the label and only experts know which natural extract or oil contains a certain allergen. This means also, that products, which are called ‘parfum free’, but contain natural substances with fragrance constituents, are not really ‘free of fragrances’, but only free of deliberately added perfume mixtures or single fragrance compounds. Some of the natural substances containing the allergenic fragrances as major constituents appear in the beginning of the ingredient lists, indicating that they are present in larger amounts compared to the single fragrance compounds added, which appear usually at the end of the ingredient lists. This shows that natural cosmetics could contain even larger amounts of the ’26 fragrance allergens’ than purely ‘synthetic’ products.

**Table 6:** Some natural substances reported in the 100 ingredient lists, which include fragrance compounds as constituents. These fragrances belong to the ‘26 fragrance allergens’

Examples	Names and percentages of fragrance constituents
*Chamomilla recucita* oil	Linalool 0.4 %, limonene 1 %
*Citrus aurantium dulcis* oil	Limonene 95 %, citral 1 %
*Citrus medica limonum* oil	Citral 3–5 %, limonene 56–78 %
*Cupressus sempervirens* oil	Limonene 5–7 %, linalool 1–3 % other manufacturer: limonene 14 %, linalool 0.8 %
*Cymbopogon martini* oil	Geraniol 66–84 %, linalool <4 %, citral <2 %, farnesol 2< %, limonene <1 % other manufacturer: limonene <22 %
*Daucus carota* oil	Linalool 1–2 %, limonene 1–2 %, citral 1–3 %, geraniol 1–3 %
*Eucalyptus globulus* oil	Limonene 7–10 %
*Juniperus communis* fruit oil	Limonene 1–3 %
*Lavandula angustifolia* oil	Limonene <1 %, linalool 40 %
*Mentha arvensis* oil	Limonene 3–4 %
*Pelargonium graveolens* oil	Citronellol 30–40 %, geraniol 12,5–15 %, linalool 7–10 %, citral 1–3 %, limonene 1–3 %
*Rosa damascena* flower oil	Citronellol 25–30 %, geraniol 20–25 %, linalool 1–3 %, eugenol 1–3 %, citral 1–3 %
*Rosmarinus officinalis* leaf oil	Linalool 0.8 %, limonene 6 %

Data derived from material safety data sheets

14 products did not contain ‘parfum’ nor ‘flavor’ nor ‘aroma’, nor even any of the ‘26 fragrance allergens’. However, it is not certain, that some of the natural substances in these products comprise allergenic fragrances as constituents.

There were no obvious differences between low- and high-priced products concerning ‘parfum’ and fragrances in the ingredient lists.

#### The data correspond to previous analysis by other authors in various countries

These findings are in line with previous analyses and show that the situation does not seem to have improved in the last years: Rastogi and his coworkers had found that 91 % of natural cosmetics on the Danish market contained at least one of the allergenic fragrances [44]. Steinemann found that the composition of volatile organic compounds in ‘green’-labeled fragranced products was not significantly different from that of other fragranced products with regard to the number of hazardous chemicals as defined under U.S. federal law [45]. Dodson described, that most common natural fragrance chemicals included the allergenic terpenes limonene, hexyl cinnamal, and linalool [27]. The frequency of the ‘26 fragrance allergens’ was analyzed also in the ingredient lists of conventional deodorants in a previous study where 76 % of the products contained at least one of the ‘26 fragrance allergens’ [46].

### Promising product names

Nine products were named after a natural ingredient, which is classified and labelled as dangerous for human health or the environment (for example ‘Lavender’ in the product name and ‘*Lavandula angustifolia* oil’ in the ingredient list, ‘Neroli’ in the product name and ‘*Citrus aurantium dulcis* flower oil’ in the ingredient list, ‘rose’ in the product name and ‘*Rosa damascena* extract’ in the ingredient list). It must be assumed that manufacturers expect that consumers do not know which natural ingredients are dangerous substances.

Furthermore, product names and ingredient lists often do not match: Three products named after a certain plant did not indicate names of these plants in the ingredient lists. For example, a soap was called ‘apricot and elder’, but there appeared no natural substance from *Prunus armeniaca* in the ingredient list.

Recommendations for sensitive skin do not always imply that the ‘26 fragrance allergens’ are omitted. Fifteen products carry the word ‘sensitive’, ‘soothing’ (‘beruhigend’), ‘soft’ (‘sanft’) or ‘for sensitive skin’ (‘für empfindliche Haut’) in their product names or subtitles. All of them contained between one and up to seven of the ‘26 fragrance allergens’. There was no obvious difference between low- and high-priced products. One product that was recommended for patients with neurodermatitis did not contain any fragrances.

Two products are called ‘vegan’, although the only substances of animal origin in this study are beeswax, honey and royal jelly extract, which are ingredients in only 25 products.

### Improvements needed

The data compilation in this paper has implications on a higher tier: it reveals examples where compliance with legal requirements needs to be improved. Some details in European chemical legislation function as waivers or are loopholes for natural substances as well as for personal care products. The data here question whether such a special treatment of natural substances and personal care products is in line with an efficient protection of human health and the environment. Table 7 is a compilation of the shortcomings with brief comments.

**Table 7:** Examples of legal requirements for natural substances and personal care products in the REACH, CLP and Cosmetics regulation illustrated by comments on the basis of the present study

	Comments
(a) Examples where compliance with legal requirements needs to be improved	
Producers do not always use the INCI names	Some substances can be identified if one makes the effort and compares the names with the descriptions of the origin in the INCI list
Many natural substances are classified differently by various notifier groups (self-classification)	Notifiers should find joint classifications. It is difficult for the external user to decide, which classifications are the correct ones. Even for CMR substances there are no harmonized classifications
Many natural substances are not catalogued in the C & L inventory although they are used in products	Data on classification and labelling and data on constituents are publically available only for a minor part of natural substances
Natural substances (being UVCB substances) must only be registered according to REACH, if they meet the criteria for classification as dangerous ([20] Art. 2(7)(b) and Annex V (8))	Many natural substances are not notified in the C & L inventory yet, therefore it is not clear whether they are or should be classified and labelled and hence registered. So far only very few natural substances classified have been registered
Manufacturers should clarify substance identities and specify all known constituents of a UVCB substance which are present above 10 % and all components which are relevant for classification with the IUPAC name, CAS number and percentage in the UVCB substance [47, 48]	Only very few natural substances comply with this requirement so far. Therefore, it is hardly possible to calculate the classification of most natural substances as mixtures according to the ECHA guidance [49]
Information should be publically available if it is ‘essential to classification and labelling,’ such as ‘the degree of purity of the substance and the identity of impurities and/or additives which are known to be dangerous’ ([20] Art. 119)	Toxicity of multi-constituent substances is dependent on the constituents. A proper assessment requires these data, but the publically availabe data are very scarce
Risk assessments of single substances should consider various discharge patterns and entry paths	However, the constituents of multi-constituent substances are usually not considered in risk assessments of the single substances (e.g. risk assessment of limonene does not include discharge of *Citrus* preparations)
The general public should be provided with safety data sheets or sufficient information for safe handling about dangerous mixtures ([20] Art. 31)	Consumers are informed only in special cases, e.g. some hair dyes about special safety arrangements. Most consumers do not expect that personal care products can be dangerous mixtures. It must be questioned whether the ingredient lists are sufficient information for safe handling
(b) Examples where natural substances and cosmetic products are granted special waivers	
Cosmetic products are not classified and labelled, even if they contain dangerous substances above the thresholds ([8] (A) Art. 1 (5))	Consumers have the right to know. This exception for cosmetic products impedes a suitable risk communication
‘26 fragrance allergens’ are only listed on the containers if they are added as single substances	Many natural substances are mixtures that contain some of the 26 fragrance allergens as constituents, which arrive ‘incognito’ in the products
Other mixtures—but not cosmetic products.—which are not classified as sensitizing but contain at least one sensitizing substance must be labelled with EUH208 ‘Contains (name of sensitizing substance). May produce an allergic reaction.’ ([8] Annex II Part 2 2.8)	Most cosmetic products, also natural products, contain sensitizing fragrances or preservatives. Only informed persons recognize them in the ingredient lists. The label EUH208 with the two short sentences would particularly be important for personal care products. Therefore this exception should be abolished
CMR substances may be used if their “use has been found safe by the SCCS” [22]	This does not correspond to the precautionary principle in the chemical legislation and to the ‘right to know’ for the consumer. There should be no exceptions for CMR substances. They should not be allowed in personal care products, even if they are natural substances
The chemical safety report does not need to consider the risks to human health from the use of cosmetic products ([20] Art. 14 5(b))	Realistic risk assessments should consider the complete sum of exposure routes. Exposure via personal care products is not negligible
Data on cosmetic ingredients need not be transmitted in the supply chain ([20] Art. 2(6b))	Manufacturers and downstream users of cosmetic products should be informed like manufacturers and downstream users of any other products. This exception should be deleted to improve transparency and risk communication
Chemical safety reports are not publicly accessible	The public availablity of chemical safety reports could improve transparency [50]
SVHCs must be authorized before they are placed on the market or used, unless they are used in cosmetic products ([20] Art. 56 (5))	With this exception for cosmetic products, authorization will affect other uses and will not enforce substitution of SVHCs in personal care products. This exception should be deleted to improve consumer and environmental protection

## Conclusions

The results question whether natural personal care products deserve the general bonus granted in the public perception. The hazards of a product depend on the properties of the ingredients and are independent whether a substance is natural or synthetic. Anthropogenic discharge of natural substances can be considerable. Therefore, natural substances deserve more attention in toxicology and ecotoxicology.

Producers of cosmetics keep affirming that their products were ‘safe’ [26]. In my understanding, it is impossible according to the rules of logic to prove that something is ‘correct’ or ‘safe’, it is only possible to describe potential risks on the basis of the present knowledge. Every application of a natural or a synthetic chemical substance goes along with a residual risk. In my opinion, manufacturers have the responsibility to reduce risks for the consumer and the environment caused by their products as far as possible, e.g. by avoiding dangerous substances in personal care products and inform appropriately about the residual risks.

Consumers should be informed that many natural substances used in personal care products are classified as dangerous substances, most of them being hazardous for skin and eyes. Consumers should also be informed that the vast majority of natural cosmetic products contain allergenic fragrance compounds, either as single compounds added or as natural constituents of various natural substances. If the consumers do not receive sufficient information about the residual risk, e.g. by classification and labelling, but receive misleading information on the containers instead, this does not correspond to the intention of the European chemical legislation which aims ‘at a high level of protection of human health and the environment.’

## Methods

The ingredient lists of 100 natural personal care products that were available on the European retail or online market were analyzed. These products were a random selection of ten products each marketed by ten established European manufacturers (German, Swiss, Danish and French) covering a wide price segment. The most expensive products were nearly 20 times as expensive as the comparable cheapest products. The purpose of this study was to make a spot check of natural products. The spectrum was as wide as possible (e.g. after shave, anti-pimples product, body lotion, cleanser, day cream, deodorant, face mask, hair color, hair conditioner, lip balm, mascara, massage oil, mouth wash, nail balm, shampoo, shower gel). The small number of products analyzed cannot be representative nor comprehensive for natural cosmetics in general. The purpose was here to compile and analyze publically available data only and prepare the floor for further studies. Natural substances were defined here as substances, which originate clearly from a specific organism (indicated by its scientific Latin binomials or known to be from a definite biological species) as described in [13]. Substances from organisms which were a chemical group of substances that had been isolated from an organism or had undergone a chemical reaction were not considered here, e.g. ‘*Brassica campestris* sterols’, ‘tapioca starch’, ‘hydrolyzed wheat gluten’, ‘hydrogenated castor oil’, or ‘hydrogenated coconut acid’. There were some borderline cases: Xanthan gum and shellac were considered here, as these substances can occur as such in nature. The natural substances were compared with the entries in the classification and labelling inventory, the so-called ‘C & L inventory’ (http://echa.europa.eu/web/guest/information-on-chemicals/cl-inventory-database). This is the official European database of classification and labelling information on notified and registered substances received from manufacturers and importers according to the CLP-regulation [8] on the basis of the REACH regulation [20] Title XI. Classification and labelling is an ongoing process, leading to a continuous update of the data. Data used have been collected from August 2013 up to August 2015. Various notifier groups classified many substances differently. It was decided to consider only the classifications effectuated by the clear majority if there was no harmonized classification.
